# Using intervention mapping for the development of a targeted secure web-based outreach strategy named SafeFriend, for Chlamydia trachomatis testing in young people at risk

**DOI:** 10.1186/1471-2458-13-996

**Published:** 2013-10-22

**Authors:** Kevin ATM Theunissen, Christian JPA Hoebe, Rik Crutzen, Chakib Kara-Zaïtri, Nanne K de Vries, Jan EAM van Bergen, Marianne AB van der Sande, Nicole HTM Dukers-Muijrers

**Affiliations:** 1 Department of Sexual Health, Infectious Diseases and Environmental Health, Public Health Services South Limburg, Geleen, The Netherlands; 2 Department of Medical Microbiology Maastricht Infection Centre (MINC), School for Public Health and Primary Care (CAPHRI), Maastricht University Medical Centre (MUMC+), Maastricht, The Netherlands; 3 Department of Health Promotion, School for Public Health and Primary Care (CAPHRI), Maastricht University Medical Centre (MUMC+), Maastricht, The Netherlands; 4 In-Fact, Bradford, UK; 5 Centre for Infectious Disease Control, RIVM National Institute of Public Health and the Environment, Bilthoven, The Netherlands; 6 The national institute for STI and AIDS Control, Amsterdam, The Netherlands; 7 Department of General Practice, AMC-University of Amsterdam, Amsterdam, The Netherlands; 8 Julius Centre for Health Sciences and Primary Care, University Medical Centre Utrecht, Utrecht, The Netherlands

**Keywords:** Intervention mapping, Chlamydia trachomatis, Screening, Partner notification, Web-based respondent driven sampling, Social networks, Sexual networks, Peer influence

## Abstract

**Background:**

Many young people at high risk for Chlamydia trachomatis (Ct) are not reached by current sexual health care systems, such as general practitioners and public sexual health care centres (sexually transmitted infection clinics).Ct is the most frequently diagnosed bacterial sexually transmitted infection (STI) among sexually active people and in particular young heterosexuals. Innovative screening strategies are needed to interrupt the transmission of Ct among young people and connect the hidden cases to care.

**Methods:**

Intervention Mapping (IM), a systematic approach to develop theory- and evidence-based interventions, was used to develop a strategy to target Ct testing towards young people who are currently hidden to care in The Netherlands. Both clinical users (i.e. sexual health care nurses) and public users (i.e., young people at risk for Ct) were closely involved in the IM process. A needs assessment study was carried out using semi-structured interviews among users (N = 21), a literature search and by taking lessons learned from existing screening programmes. Theoretical methods and practical applications to reach high risk young people and influence testing were selected and translated into specific programme components.

**Results:**

The IM approach resulted in the development of a secure and web-based outreach Ct screening strategy, named SafeFriend. It is developed to target groups of high-risk young people who are currently hidden to care. Key methods include web-based Respondent Driven Sampling, starting from young Ct positive sexual health care centre clients, to reach and motivate peers (i.e., sex partners and friends) to get tested for Ct. Testing and the motivation of peers were proposed as the desired behavioural outcomes and the Precaution Adoption Process Model was chosen as theoretical framework. End users, i.e., young people and sexual health care nurses were interviewed and included in the development process to increase the success of implementation.

**Conclusions:**

IM proved useful to develop an intervention for targeted Ct testing among young people. We believe this to be the first web-based outreach screening strategy which combines chain referral sampling with the delivery of targeted Ct testing to high risk young people within their sexual and social networks.

## Background

Testing and treating are essential strategies in Chlamydia trachomatis (Ct) control to interrupt the inherent transmission chain [1]. In many countries, including the Netherlands, positivity rates of Ct have increased in the past years [2-4]. Ct is thereby the most frequently diagnosed bacterial sexually transmitted infection (STI) among sexual active people and in particular young heterosexuals. Young people below 25 years of age comprise a major risk group for Ct control, because they show constant high rates of sexual risk behaviour and potentially bear the largest burden of STI sequelae in terms of reproductive morbidity [5].

Web-based Ct screening programmes have improved the access to sexual health care beyond the sexual health care centre and general practitioners (GPs) by engaging young people in discussions about sexual health, increasing home-based testing, and enhancing the management of Ct patients and re-screening positives [6-9]. Unfortunately, the success of such early initiatives was limited in the Netherlands (e.g., Chlamydia Screening Implementation Program “CSI”) and in other counties [8,10,11]. One of the reasons for this was that such initiatives did not readily reach the targeted high-risk young people [8,10,11], leaving this group largely hidden to health care providers. Therefore there is an urgent need to find new ways to tap into such hidden key populations and find a natural way of motivating them to access sexual health care [12]. To clarify the concept, a key population for a given infection consists of members who are either infected or have a very high risk of acquiring the infection. Reason for having (high risk for) infection is the membership to high risk sexual and social networks. Typically, infections thrive in such networks which provide the right vehicle for transmission of the infection taking advantage of the inherent risky behaviour and social proximity [13,14]. Currently, a significant number of members of such network remain completely invisible to care (hidden key population).

Members of the social and sexual networks surrounding Ct positives typically showed high risk [15,16]; these networks are important targets for interventions [17]. Information about STI can be readily spread within a social network and thereby influence risk behaviour such as unprotected sex practices [15,17]. Furthermore, networks can overlap whereby a social network creates a venue to meet new sex partners [15,17]. There is now a growing body of evidence on the high success potential of network based interventions, e.g. by using chain referral sampling methods such as web-based Respondent Driven Sampling (web-based RDS) and peer influence to reach hitherto hidden networks [18]. The basic idea behind RDS is that selected young people are recruited as seeds or ambassadors who themselves motivate others in their own networks to get tested, and so on. The RDS methodology has already proven its worth in other public health areas (e.g., health promotion) and target populations (e.g., injection drug users and HIV patients) [19-21].

This paper describes the development of an innovative secure web-based outreach Ct screening strategy, whereby young people at high risk for Ct (re)-infection will be targeted and encouraged to go for testing. The project is carried out by one of the eight coordinating public sexual health care centres (STI clinics) in the Netherlands, where the intervention will be developed, tested and implemented. The objective is to develop a cost-effective and sustainable web-based outreach screening strategy for reducing the patient’s and population’s Ct burden, and improving the timing of care. The Intervention Mapping (IM) protocol can be used to guide the systematically development of the intervention based on theory and evidence, which should increase the likelihood of developing an effective intervention [22]. IM has already been applied in different populations and study areas such as drug users, condom use, and stress related mental disorders [23-25], but never in the development of Ct screening programmes among high risk young people.

## Methods

### Step 1: Needs assessment

The Intervention Mapping process starts with a need assessment to analyse a specific health problem within a target population and associated important stakeholders. Furthermore, behavioural and environmental risk factors that contribute to the health problem are identified and the underlying determinants are explored. At the end the programme outcomes are stated. To plan and guide the need assessment the PRECEDE-model is used [26].

**Figure 1:**
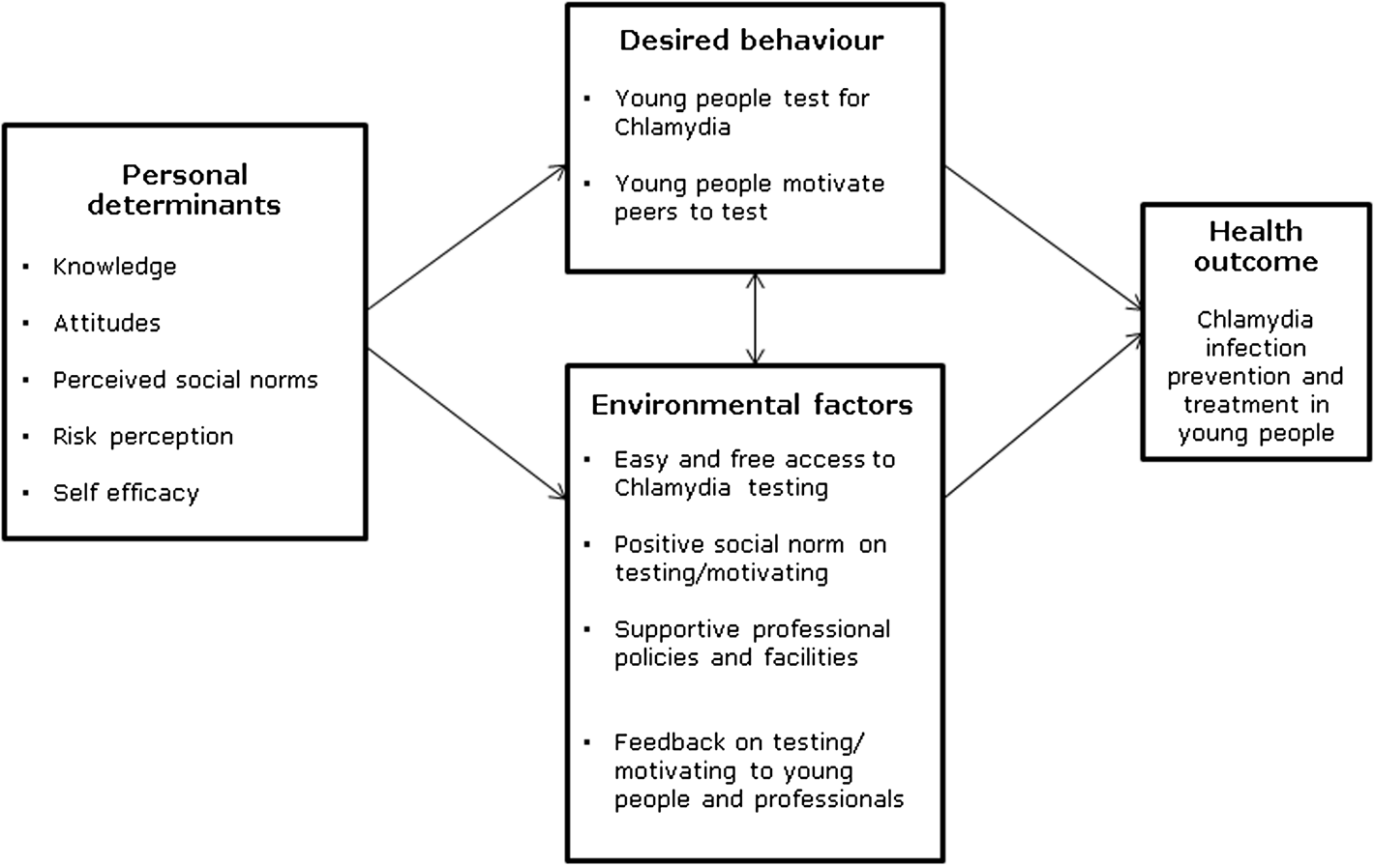
Needs assessment using a simplified PRECEDE model (see Methods and Tables).

The Dutch national STI policy targets were explored in order to identify expectations from practical and political stakeholders. A literature search was conducted reporting on Ct prevalence in different population groups, including young people, and existing strategies for Ct control, why these strategies failed or succeeded, and which methods were cost-effective to reach high risk young people. Furthermore, semi-structured interviews were held among Ct positive young people (8 women and 13 men) who were between 17 and 24 years old and visited the sexual health care centre South Limburg for Ct treatment. They were from various ages, sexual orientation, educational levels and ethnicity. They were interviewed about partner notification (PN) to find out the determinants to motivate peers to test for Ct. Ethical approval of the Regional Medical Ethics committee in the Netherlands was not necessary, because participants in these interviews were not “subjected to procedures or required to follow certain rules of behaviour” [27]. Based on the needs assessment using the PRECEDE-model (Figure 1), the health problem, behavioural and environmental factors, the underlying determinants and the target group were defined, resulting in two desired behavioural outcomes that were selected to be targeted by the intervention.

### Step 2: Preparing matrices of change objectives

In step 2, each of the behavioural outcomes is broken down into performance objectives, i.e. the actions that expectedly lead to the desired outcome. For each performance objective, the most important and changeable determinants are stated based on theoretical models for behavioural change. Combining the performance objectives with the determinants identified results in matrices that include the change objectives that guide the intervention development process.

The performance objectives and most important and changeable determinants were selected based on previous studies, the results of the semi-structured interviews and the Precaution Adoption Process Model (PAPM) [28]. The model comprises of a stage theory that ranges from unaware of a problem to action and maintenance of a specific behaviour (e.g., Ct testing and motivating network members to test). This model was chosen, because it emphasizes that hazard responses are influenced by behaviours and opinions of peers, which is in line with the underlying of social and sexual networks surrounding Ct positives. A person’s decision concerning the need to test is influenced by the opinion and actions of peers and not just by specific beliefs about the hazard that lead to rational and independent decisions. This social influence may occur at several stages of the model.

### Step 3: Selecting theory-informed intervention methods and practical applications

In step 3, theory-informed intervention methods are selected to create changes in the determinants of the behavioural outcomes. The relevance of methods for a particular project and the changeability that can be realized with a particular method guides this selection process. Practical applications that fit the target population and context are then translated from these theory-informed intervention methods.

Before a final list of methods was determined on the basis of existing evidence and theory, theoretical methods were explored and analysed by a small panel of experts consisting of researchers, prevention experts, informatics-specialists, nurses and medical doctors. The experts then transformed the methods into practical applications taking into account the parameters of use, the literature review and conducted semi-structured interviews.

### Step 4: Producing program components and materials

In this step, the practical applications are combined and translated into programme components and specific materials. During this process several end users are included to select the best program design that fits the user’s preferences. The intervention is then pilot-tested among some members of the target population. Eventually, necessary adjustments are made and the definitive intervention developed.

The intervention was constructed, tested and refined with close involvement of high risk young people and sexual health care nurses. The selected applications were incorporated into two websites: (1) a smart and highly interactive website for young people to communicate and motivate those at high risk in their social and sexual networks and (2) another website for sexual health care professionals to start recruitment-chains and oversee the process of home-test requests. The final name and look-and-feel of the intervention were set after pre-testing it among young people. The first pre-test of the intervention will be conducted among young people (age ≤ 25 years) visiting the public sexual health care centre South Limburg for Ct treatment. Evaluation will then be done to identify facilitating factors and barriers to use and the application is refined further (specific guidance for graphic designers and programmers).

The last two steps of the IM protocol, step 5 and 6, are outside the scope of this paper and are described briefly here.

### Step 5: Planning program adoption, implementation, and sustainability

An implementation plan is made to deploy the intervention to a successful outcome. End users and implementers of the intervention are identified and implementation objectives are stated considering dissemination, adoption, implementation and maintenance of the intervention.

Health care professionals (e.g., sexual health care nurses, medical doctors) and policy makers were involved from the start of the project by regular team meetings. They are the key linkage group for the success of the development and implementation and have therefore been involved in the entire research process. The ultimate goal is national implementation in sexual health care centres and the future expansion to GPs will be explored.

### Step 6: Planning for evaluation

In the final step of intervention mapping, an evaluation plan and associated criteria are set up. The level of evaluation is chosen and indicators and measures determine to evaluate the effects and process of the intervention. All program objectives are defined in a measurable form.

To assess whether the intervention leads to changes in determinants and thereby in a change in behaviour of young people (i.e., Ct testing and motivate high risk young people to test for Ct) a plan for the quantitative and qualitative evaluation was made. Factors that may hinder or facilitate the use of the intervention will be reviewed in the course of this project and strategies will be explored to positively affect intervention use.

## Results

### Step 1: Needs assessment

#### Health problem and target group

The needs assessment started with a literature search and the exploration of national stated aims which revealed that young people below 25 years of age comprise a major risk group for Ct control, because they show constant high rates of sexual risk behaviour and potentially bear the largest burden of STI sequelae in terms of reproductive morbidity [5]. Every year, there is a large new accretion of about 200,000 susceptible sexually active Dutch young people, although not all are at high risk for acquiring Ct [1]. Estimates about the total burden of Ct in 15-30-year-olds suggest 60.000 new infections yearly [29], with an estimate of only 30.000 infections diagnosed in sexual health care centres and by GPs [Personal communication dr. I van den Broek, based on available data from STI centres and GP-surveillance, [2,30]. Hence, half of the Ct infections remain undetected and hidden to care and there is an urgent need to significantly increase Ct testing rates among young people at high risk. Young people receive and share information about STI and associated testing through their peers, i.e. a combination of sex partners and friends [17,31]. Their behaviours, attitudes and actions in respect to STIs are strongly influenced by their peers who are therefore key change agents [17,32]. There is clear evidence of high STI/Ct prevalence in social networks surrounding STI positive people [15,16,33,34]. Such networks thrive on high behavioural risk and therefore provide a rich conduit for rapid STI spread and re-infection [16,17]. Based on the health problem and selected target group two desired behavioural outcomes were formulated: a) High risk young people (≤25 years of age) get tested for Ct, and b) motivate peers via their social and sexual network to get tested.

### Determinants for Ct testing and motivating peers to test

Several behavioural barriers for testing among young people have been identified from the literature search (i.e., Table 1). For example the level of knowledge of STI; Ct being an asymptomatic disease, the connection between Ct and fertility complications and current testing options [35-37]. Another barrier includes misperceived vulnerability; young people do not think they are at risk for Ct [35,36,38]. Embarrassment about the need for testing and fear of a positive test result also plays a role [36,38,39]. Shame and fear may be enforced when the provider is a family doctor and young people are charged for testing (note that Dutch sexual health care centres test free of charge) [37-39]. Home-based test were found to be acceptable among young people and could be used as a facilitator factor in testing [9,39-41].

**Table 1:** Determinants for Chlamydia (Ct) testing and motivating peers for Ct testing among young people by systematic literature search (March 2012) and semi-structured interviews (n = 21)

**Ct Testing**	**Motivating peers**
*Personal determinants*	*Personal determinants*
Knowledge about:	Knowledge about the transmission of Ct among sexual and social network members [interviews]
- STI and in particular asymptomatic disease [35-37]	
- Ct effects [36,37,39]	
- Ct testing methods [36,37]	
Fear of parental notification [36]	
Attitudes towards testing:	Attitudes towards motivating:
- Shame about Ct testing [37-39]	- Perceived stigmatization/embarrassment [42-44], interviews
- Embarrassment about Ct testing [36-39]	
- Fear of test results [36,38,39]	- Emotional commitment with peers [42,44], interviews
- Fear of testing procedure [37,39]	- Personal health benefit (re-infection) [42], interviews
	- Blaming towards sexual partners [42]
	- Fear of reaction (e.g. aggression) [42-44], interviews
Perceived social norms of Ct testing among peers [38,39], interviews	Perceived social norm of motivation among peers [interviews]
Risk perception towards testing:	Availability and accessibility of contact details*
- Awareness of vulnerability/risk for Ct	
[35,36,38]	
	[42-44], Interviews
- Awareness of severity of Ct [36,38]	
Self-efficacy:	
- Afraid to visit public health services [37], interviews	

* will be excluded in this study, because this determinant can not be changed.

Barriers related to motivate peers to get tested for Ct include fear of stigmatization among peers, repercussion (e.g. aggression), blaming the other, availability of contact details and the level of emotional commitment [38,42-44].

The analyses of the 21 semi-structured interviews showed several barriers and facilitators related to motivating peers to get tested for Ct. Ct positive young people can feel embarrassed about their positive Ct test result when motivating peers to get tested. Some indicated that in case of a casual sex partner they would like to notify anonymously. Fear of aggression of sex partners can also discourage Ct positive young people to motivate peers. Interviewees expressed emotional commitment to their sex partners and felt responsible for informing them about the Ct risk. They were aware of the possible health risks for direct and indirect sex partners, whereas others were afraid of re-infection. As stated by one interviewee: *“Partner notification is self-evident, when you do not warn your steady partner, you will get re-infected”.* Social influence plays also an important role. Several interviewees were notified about their Ct risk via their sex partners and then visited a sexual health care centre. They also indicated that friends visited a sexual health care centre after talking to them about Ct testing and their own positive test result. As noted by one interviewee: *“We [friends] talk about everything. We do not have any problems to talk about Ct positive test results”.*

### Step 2: Preparing matrices of change objectives

For each of the 2 behavioural outcomes 4 performance objectives were defined (i.e., Tables 2 and 3). Firstly, young people should be able to appraise their own and others Ct risk behaviour. Secondly, if necessary, young people should decide to get tested and motivate peers who are at risk for Ct to get tested. Thirdly, planning for getting tested or motivating peers is another objective which will lead to the fourth objective of actually getting tested and motivating other young people at risk for Ct. The following determinants were identified as most important and changeable: knowledge, self-efficacy, risk perception, social norms and attitudes. Finally, a matrix of change objectives was developed for each behavioural outcome.

**Table 2:** Performance and change objectives for the behavioural outcome: high risk young people get tested for Chlamydia (Ct)

**Determinants**					
**Performance objectives**	**Knowledge**	**Attitude**	**Perceived social norms**	**Risk perception**	**Self-efficacy**
PO1.1: Young people appraise effects of Ct and personal risk	K1.1: Describe different STI and in particular asymptomatic diseases	A1.1: Express positive attitude towards self assessment of Ct risk		R1.1: Aware of the possibility of getting a Ct infection	
	K1.2: Describe Ct effects				
PO1.2: Young people decide to get tested for Ct	K1.3: Describe test procedure and test options for Ct			R1.2: Aware of the health risk of not getting tested	
PO1.3: Young people ask for Ct testing					SE1.1: Express confidence in ability to ask for testing option (home based test kits or appointment STI centre)
PO1.4: Young people get tested for Ct		A1.2: Express positive attitude towards test procedure and results.	SN1.1: Recognize social acceptance of Ct testing among young people		

**Table 3:** Performance and change objectives for the behavioural outcome: young people motivate peers to get tested for Chlamydia (Ct)

**Determinants**			
**Performance objectives**	**Knowledge**	**Attitude**	**Perceived social norms**
PO2.1: Young people appraise peers to get tested	K2.1: Recognize the risk of Ct among social and sexual network members		
PO2.2: Young people decide to motivate network members for testing		A2.1: Feel responsible to motivate high risk young peers	
PO2.3: Young people plan to motivate peers		A2.2: Express positive attitude towards the motivation of peers to get tested	
PO2.4: Young people motivate peers			SN2.1: Recognize social acceptance among young people to motivate peers at high risk for Ct

The treatment and follow up for re-testing is already included in the current sexual health care practice and is therefore not described within the matrices.

### Step 3: Selecting theory-informed intervention methods and practical applications

As a result from the initial needs assessment and the matrices of change in step 2, a web-based RDS was used to select peers to motivate young people and encourage them to attend Ct testing, thereby motivating high-risk young people through their own social and sexual network. Web-based RDS was judged highly promising and feasible in high-risk young people, because it has been successfully used before in a group of young people [18]. The concept of using peer influence and chain-referral sampling has already worked well to target “those in need” in complex networks but never used in Ct control in young people [19,45,46]. In our strategy sampling begins with Ct positive young people attending the South Limburg sexual health care centres and proceeds in waves recruiting more targeted contacts from hitherto-hidden sexual and social networks. Participants select friends based on their knowledge of the extent to which these friends need or use appropriate care. The person who begins the chain-referral sampling within a given high risk network is already tested for Ct, which increases perceived social norms. To improve the chance of the targeted contact to decide for testing, messages and home-based test kits will be used. Other supporting methods geared to improve testing and motivating include tailoring, personalize risk, modelling, consciousness raising, elaboration, mobilizing social support and self re-evaluation (i.e., Tables 4 and 5).

**Table 4:** Methods and applications to get high risk young people tested for Chlamydia (Ct)

	**Methods**	**Parameters for use**	**Applications**
*Knowledge*			
K1.1: Describe different STI and in particular asymptomatic diseases	-Tailoring	-Tailoring variables or factors (e.g., socioeconomic status)	AP1.1: Online tailored information about STI based on current knowledge and Socioeconomic Status (SES)
	-Elaboration	-Individuals with high motivation, messages that are personally relevant and easily understandable.	AP1.2: Information about the different Ct test options and procedures using videos and images.
K1.2: Describe Ct effects			
K1.3: Describe test procedure and test options for Ct			
*Attitude*			
A1.1: Express positive attitude towards self assessment of Ct risk	-Self-re-evaluation	-Stimulation of both cognitive and affective appraisal of self-image	AP1.3: Stimulation via visual effects (videos, and images) and questions to self assess sexual behaviour.
	-Modelling	-Attention and identification with model	AP1.4: Messages from friends/sex partners to motivate young people to self assess their sexual behaviour
A1.2: Express positive attitude towards test procedure and results.	-Elaboration	-Individuals with high motivation, messages that are personally relevant and easily understandable.	AP1.2: Information about the different Ct test options and procedures using videos and images.
*Perceived social norms*			
SN1.1: Recognize social acceptance of Ct testing among young people	-Modelling	-Availability of social and sexual network	AP1.5: Via personalized or anonymous messages from peers young people are encouraged to get tested for Ct. Information about Ct test options and informs that a friend or sex partner already asked for a Ct test.
	-Mobilizing social		
	-Support		
*Risk perception*			
R1.1: Aware of the possibility of getting a Ct infection	-Personalize risk	-Messages are personal and results are compared to absolute and normative standards.	AP1.6: Risk assessment questionnaire will be provided and a personalized report about the acquired Ct risk and an advice about Ct testing is provided to the person.
	-Consciousness		
			AP1.7: Reminders will be send to young people who did not yet ask for a Ct test.
	-Raising	-Can use feedback	
R1.2: Aware of the health risk of not getting tested			
*Self-efficacy*			
SE1.1: Express confidence in ability to ask for testing option (home based test kits or appointment STI centre)	-Planning coping responses	-Identification of potential barriers and solutions	AP1.8: Young people will be informed about the possibility and advantages of home-based Ct test kits. These test kits are free, anonymous and easy to use.

**Table 5:** Methods and applications for young people to motivate peers to get tested for Chlamydia (Ct)

	**Methods**	**Parameter for use**	**Applications**
*Knowledge*			
K2.1: Recognize the transmission of Ct among social and sexual network members	-Tailoring	-Tailoring variables or factors (e.g., socioeconomic status	AP2.1: Online information about the transmission of Ct among social and sexual network via videos and images.
	- Elaboration	-Messages that are personally relevant, easily understandable and include direct instructions	
*Attitude*			
A2.1: Feel responsible to motivate high risk young peers	-Elaboration Modelling	-Messages that are personally relevant, easily understandable and include direct instructions - Attention and identification with model	AP2.1: Online information about the transmission of Ct among social and sexual network via videos and images. AP2.2: Via personalized or anonymous messages from peers young people will be encouraged to motivate their own friends/sex partners.
A2.2: Express positive attitude towards the motivation of peers to get tested			
*Perceived social norms*			
SN2.1: Recognize social acceptance among young people to motivate peers at high risk for Ct	-Modelling	-Attention and identification with model	AP2.2: Via personalized or anonymous messages from peers young people will be encouraged to motivate their own friends/sex partners.
	- Mobilizing social support	-Availability of social and sexual network	

### Step 4: Producing program components and materials

As a result of the selected methods and practical applications the deliverable will be an outreach Ct screening strategy based on a secure web-based RDS engine, with an online interface specially written for young people to recruit and motivate peers. This website is named http://www.SafeFriend.nl. The use of the web was judged promising during this IM process, because many young individuals have an email address and/or mobile phone to reach them. Using messages via email and/or mobile phone young people can motivate their sex partners and/or friends. The message will be brief and contains carefully worded text to alert the receiver of possible Ct risk, provide options for Ct testing and modelling (i.e., peers who test). To increase the likelihood of use the exact content of this message can be partly adapted (i.e., name or anonymous, level of language) by the user who sends it. After login and system user verification the person is offered tailored information on sexual health and likely Ct risk. They will be prompted with a brief questionnaire and the answers are automatically assessed to decide to offer him or her a home-based Ct test. Home-based testing has proven feasible and effective in other screening programs, however, such internet-delivered testing is yet to be included in regular care in public sexual health care centres or other care settings. The new recruit is then encouraged to motivate at that moment or at a later stage others who will then go through the same procedure. It should be noted that within the website in some cases a young individual will be asked to visit the sexual health care centre as they are likely to require more sexual health care than Ct testing alone. Necessary facilities like computers and home-based Ct test kits will be in place at the moment the intervention is implemented in practice. Overall, the use of the SafeFriend website by young people is self-initiated and not guided by the care professionals. The exception is the first recruitment step where the Ct positive young people at the sexual health care centre are motivated and supported by the nurses to use the intervention. For this, a second designed interface is accessed by the nurse during the consultation visit (the moment that in routine care PN is discussed). This interface enables the nurse to start the recruitment-chain and to oversee the process of further home-test requests. The young Ct positive client can motivate others during the consultation (like in provider referral) and/or at a later moment at home (like in patient-referral). Of course the clients may choose not to motivate others, without any further consequences for their own care. As the actions on motivating others are standardly registered (coded) the intervention yields systematic data on PN, data which are currently lacking.

## Discussion

Intervention Mapping has been used to develop a web-based outreach strategy, named SafeFriend, for Ct testing in young people at risk. The intervention will be used to target high risk young people as opposed to the current wider web-based Ct screening strategies which have used a more opportunistic [6,9] or population approach [8,10]. It is believed that the current research project is the first web-based outreach screening strategy that combines a web-based RDS method with the delivering of targeted Ct screening to high risk young people using peer influence, starting from young Ct positive sexual health care centre clients. The sexual health care setting acts as a sustainable source of Ct positive young people and thereby provides continued access to more hard-to-reach group of high-risk young people in their networks. Specifically, this strategy aims to extend Ct prevention beyond sexual networks by also recruiting high-risk young people within social networks.

The attitudes and behaviours of young people in respect of sexual behaviour and STIs are strongly influenced by their peers [15,17,32]. Peer influence regarding STI screening is therefore an important strategy to reach high risk young people for STI testing. The Ct positive young people, who begin the web-based chain referral sampling in the proposed intervention, will have experiences with Ct testing and treatment. Their specific knowledge and positive attitudes about Ct testing could make them ideal peers to communicate messages within their sexual and social networks. However, the reliance on the success of motivation and engaging peer networks using peer influence could be reason for some concern. Personal and social sensitivities regarding STI (i.e., stigmatization, confidentiality and privacy) can be important barriers and can counter the success of peer influence. To ensure medical confidentiality and provide a secure web-based screening strategy, we enable persons to decide for themselves which personal information they reveal to their own chosen sex partners and/or friends; personal information will, in all cases, be completely invisible to all other peers. Having said this, STI is a sensitive and personal topic, especially if a STI is diagnosed, still other studies have found that youngsters did share their results with peers (i.e., 93% in CSI) [31].

The use of online Ct testing of larger populations and associated home-based test kits is increasing worldwide and proved already feasible and acceptable among young people [7,9,40,41,47]. It creates easy access to testing and overcomes barriers such as the time required for travelling, transportation and fear of anonymity loss [48]. Despite these advantages, the cost per case detected and treated is high due to low participation and Ct positivity rates [8,10], caused by targeting sexual networks only (i.e., thereby missing non-sexual high-risk contacts) or by taking a population based approach (i.e., including lower risk individuals). The developed strategy, is expected to be more targeted and cost-effective for Ct control starting from high risk young people (i.e., Ct positive sexual health care clients) and also including social networks (i.e., non sexual high-risk individuals).

In addition, the intervention should increase re-infection control by facilitating also web-based PN. Using a web-based RDS method will respectively reduce the delay of sex partner’s testing and increases the number of detected Ct infections among sex partners and friends [41]. For an actual reduction of Ct transmission and re-infection effective Ct treatment is necessary. In two previous studies, more than 90% of sex partners and positive patients being tested with home-based test kits received treatment [40,41].

Although web-based PN systems are emerging [43,49], comprehensive evaluations to determine the effectiveness of these systems are limited: data regarding socio-demographics and actual outcomes (i.e., testing and treatment of partners) are not systematically collected. To obtain more insight into infection patterns and partner management, sexual and social contacts should be linked with socio-demographics at the individual and network level. The proposed intervention will yield systematic data on infections patterns and PN. The methodology in this paper (i.e., web-based RDS) in combination with social network techniques can structurally identify network clusters and the socially prominent characteristics associated with the clusters. Identifying and understanding the boundaries and bridges within a network will also be possible and can provide useful information about the extent of network penetration. Furthermore, available data show that current PN practices, which are mainly by patient referral [50,51], could be improved significantly when there is more provider-oversight and support [34,42,47], a strategy that is also accommodated in our intervention.

The intervention will be developed, tested and implemented within a public health care service involved in STI control. An important pillar of public healthcare is the support of vulnerable groups in society. If a patient is less capable to warn his/her partner, due to low self-efficacy or misperceived severity, public health care professionals may be inclined to take responsibility in the interest of both the patient and his/her sex partner (i.e., prevention and re-infection control). However, tension exists between achieving benefits for whole populations and protecting the individual’s rights [52]. The intervention therefore facilitates both patient and provider referral, creating a digital environment where clients can voluntarily, but with the support and oversight of sexual health care nurses motivate peers to get tested.

## Conclusion

In conclusion, IM was a useful tool in developing an intervention for targeted Ct testing among high risk young people. The proposed intervention SafeFriend will be the first web-based outreach Ct screening strategy for high risk young people which includes web-based chain referral sampling to motivate peers (i.e., sex partners and fiends) to get tested for Ct. The intervention will complement existing sexual health care, by introducing the Internet to targeted Ct screening. It builds on lessons learned form a large-scale Dutch population based Ct screening programme using home-based test kits and adds to current initiatives to strengthen web-based PN. We hope it can become an integral part in sexual health care for reaching high risk populations with Ct screening and treatment which is important for both the individual and public health level.

## Abbreviations

Ct: Chlamydia trachomatis; GPs: General practitioners; IM: Intervention mapping; PAPM: Precaution adoption process model; PN: Partner notification; STI: Sexually transmitted infection.

## Competing interests

The authors declare that they have no competing interests.

## Authors' contributions

KT carried out the study, participated in the design of the study, performed the analyses and drafted the manuscript. ND supervised the study, designed the study and drafted the manuscript. CH, RC, CKZ, NV, MS and JB participated in the design of the study and helped draft the manuscript. CH, RC, CKZ and NV further importantly contributed to interpretation of results. All authors read and approved the final manuscript.

## Pre-publication history

The pre-publication history for this paper can be accessed here:

http://www.biomedcentral.com/1471-2458/13/996/prepub
